# Conservative nonsurgical knowledge of German orthopedic and trauma surgeons—Present state of affairs

**DOI:** 10.1007/s00132-024-04564-w

**Published:** 2024-09-16

**Authors:** Yasmin Youssef, Rene Toussaint, Jörg Ansorg, Götz Dimanski, Dominik Adl Amini

**Affiliations:** 1 Department of Orthopedic, Trauma and Plastic Surgery, University Hospital Leipzig, Liebigstr. 20, 04103 Leipzig, Germany; 2 Praxis für Orthopädie und Sportmedizin am Brühl, Leipzig, Germany; 3 Berufsverband für Orthopädie und Unfallchirurgie (BVOU e. V.), Berlin, Germany; 4 RehaZentrum Bremen, Bremen, Germany; 5 Center for Musculoskeletal Surgery, Charité – Universitätsmedizin Berlin, Corporate Member of Freie Universität Berlin and Humboldt-Universität zu Berlin, Berlin, Germany

**Keywords:** Non-surgical treatment, Conservative treatment, Musculoskelettal disorders, Non-surgical skills, Manual medicine, Nichtchirurgische Behandlung, Konservative Behandlung, Muskuloskelettale Erkrankungen, Nichtchirurgische Fähigkeiten, Manuelle Medizin

## Abstract

**Background:**

After the fusion of the fields of orthopedics and trauma surgery in 2006 the educational content significantly increased. The acquisition of non-surgical diagnostic and treatment skills seems to fall behind in the classical operatively focused residency programs. This study presents a status quo of the non-surgical education and knowledge in the field of orthopedics and traumatology in Germany.

**Methods:**

An online-based voluntary and anonymous questionnaire was conducted between June and August 2023. The questionnaire was distributed through the email lists of the German Society for Orthopedics and Traumatology (DGOU) and the German Professional Association for Orthopedics and Traumatology (BVOU).

**Results:**

A total of 486 German orthopedic and trauma surgeons answered the online questionnaire (77.9% male; mean age 50.2 ± 11.8 years) and 11.5% were residents. Only 27.1% spent part of the residency training in the outpatient sector. In total 84.2% wish for an increased focus on non-operative treatment options during further education, 81.1% agreed that they have a good general understanding of non-operative treatment options and 81.0% felt confident to apply them in the daily clinical routine (residents 35.4% and 41.7%, respectively). The highest self-assessed competences were knowledge on the application of splints and casts and physiotherapy, ergotherapy and sports therapy, the lowest were knowledge on acupuncture, magnetic field therapy and nutritional aspects after trauma. In total, 77.7% stated non-surgical research projects are not supported at their institution.

**Conclusion:**

Orthopedic and trauma surgeons in Germany subjectively have solid knowledge on treatment options while resident physicians still need to strengthen their skills. Rotation into the outpatient sector and rehabilitation facilities as well as supporting research in the field could further improve the non-surgical skills.

## Abstract (de)

**Hintergrund:**

Nach der Fusion der Fachgebiete Orthopädie und Unfallchirurgie im Jahr 2006 haben die Ausbildungsinhalte deutlich zugenommen. Der Erwerb von nichtchirurgischen Diagnose- und Behandlungskompetenzen scheint in den klassisch operativ ausgerichteten Weiterbildungsprogrammen ins Hintertreffen zu geraten. Diese Studie stellt den Status quo der nichtoperativen Ausbildung und der Kenntnisse im Fach Orthopädie und Unfallchirurgie in Deutschland dar.

**Methoden:**

Es wurde eine onlinebasierte, freiwillige und anonyme Umfrage wurde zwischen Juni und August 2023 durchgeführt. Der Fragebogen wurde über die E‑Mail-Listen der Deutschen Gesellschaft für Orthopädie und Unfallchirurgie (DGOU) und des Berufsverbands für Orthopädie und Unfallchirurgie (BVOU) verteilt.

**Ergebnisse:**

Insgesamt 486 deutsche Orthopäden und Unfallchirurgen beantworteten den Online-Fragebogen (77,9 % männlich; Durchschnittsalter 50,2±11,8 Jahre), 11,5 % waren Assistenzärzte. Nur 27,1 % verbrachten einen Teil der Facharztausbildung im ambulanten Sektor. Insgesamt wünschen sich 84,2 % einen stärkeren Fokus auf nichtoperative Behandlungsmöglichkeiten in der Weiterbildung, 81,1 % stimmten zu, dass sie ein gutes allgemeines Verständnis für nichtoperative Behandlungsmöglichkeiten haben, und 81,0 % fühlten sich sicher, diese im klinischen Alltag anzuwenden (Assistenzärzte 35,4 % bzw. 41,7 %). Die höchsten selbst eingeschätzten Kompetenzen waren Kenntnisse über das Anlegen von Schienen und Gipsverbänden sowie über Physiotherapie, Ergo- und Sporttherapie, die niedrigsten Kenntnisse über Akupunktur, Magnetfeldtherapie und Ernährungsaspekte nach einem Trauma. Insgesamt gaben 77,7 % an, dass nichtchirurgische Forschungsprojekte in ihrer Einrichtung nicht unterstützt werden.

**Schlussfolgerung:**

Die Orthopäden und Unfallchirurgen in Deutschland verfügen subjektiv über ein solides Wissen bezüglich Behandlungsmöglichkeiten, während die niedergelassenen Ärzte ihre Fähigkeiten noch ausbauen müssen. Eine Rotation in den ambulanten Sektor und in Rehabilitationseinrichtungen sowie die Unterstützung von Forschung auf diesem Gebiet könnten die nichtchirurgischen Fähigkeiten weiter verbessern.

## Introduction

In Germany the departments of orthopedics and traumatology were merged in 2006. While the length of the residency programs remained at 6 years the educational content significantly increased [1–3]. Orthopedics and trauma surgery involves the detection and management of acute and chronic as well as acquired and congenital conditions across all age groups [4, 5]. Here, both non-surgical and surgical treatment strategies are possible for achieving an adequate outcome [4, 5].

With an increasing complexity and subspecialization of orthopedics and traumatology in Germany, a point of concern is that the whole spectrum of orthopedics and trauma surgery, including diagnostic, treatment and rehabilitation measures, cannot be covered during residency [1–3, 6]. The non-surgical diagnostic and treatment strategies seem to fall behind in the classical operatively focused residency programs. This is favored by the fact that most of the residents in Germany spend the complete residency time in a clinical and not an outpatient setting [1].

As there are no objective data on the status of the non-surgical education and knowledge of German orthopedic and trauma surgeons, the aim of this study was to present a status quo of the scope of non-surgical education and knowledge in the field of orthopedics and traumatology in Germany.

## Methods

### Study design

A nationwide cross-sectional online survey was conducted to assess the status of conservative non-surgical knowledge among orthopedic and trauma surgeons in Germany. The study was provided as an online survey with the use of a commercially available product (SurveryMoneky Inc.; San Mateo, CA, USA; https://www.surveymonkey.com). The survey was distributed via email by the German Society for Orthopedics and Traumatology (DGOU) and the German Professional Association for Orthopedics and Traumatology (BVOU) and was open between June and August 2023. Participation was voluntary and anonymity was guaranteed. By answering the questionnaire, the respondents gave consent for data collection, processing and use for publication. The study was reviewed and approved by an institutional ethics committee (#EA4/097/23).

### Questionnaire

The questionnaire was developed in four phases. In the first phase the authors YY, RT, GD, and DAA brainstormed aspects of interest about the current state of non-surgical education in Germany. In the second phase a first version of the questionnaire was drafted by the author YY. In the third phase the authors YY, RT, GD and DAA reviewed and shortened the first draft. The preliminary questionnaire was then pretested among independent orthopedic and trauma surgeons and was finalized considering the feedback from this pilot group. The final questionnaire consisted of 38 variables in 5 sections: a) sociodemographic data, b) current competences in the manual evaluation of different body regions, c) current understanding of non-operative diagnostic and treatment tools/methods, d) the scope of non-operative education experienced and e) requests concerning non-surgical education. In sections b) and c) a 5-point Likert-scale was used, the response options being “I totally agree” (corresponding to 1 on the scale), “I rather agree” (0.75), “neutral” (0.50), “I rather disagree” (0.25) and “I totally disagree” (0).

### Data analysis

Statistical analysis was performed using Excel 2019 Version 16.53 (Microsoft Corporation, Redmond, WA, USA) and SPSS (SPSS for Mac, version 26.0, Chicago, IL, USA). Categorical data were presented in frequencies (*n*) and percentages (%) and continuous data were described as means and standard deviations (SD). In addition to a total analysis of all respondents, residents were analyzed separately.

## Results

A total of 486 German orthopedic and trauma surgeons answered the online questionnaire. Overall, 77.9% (374/480) were male and 21.9% (105/480) were female. The mean age was 50.2 years (SD: ±11.8 years). In total 11.5% (55/478) of the respondents were residents, 34.1% (163/478) were specialists, 27.6% (132/478) consultants and 26.8% (128/478) senior consultants.

Overall, 29.4% (142/483) worked in the outpatient setting, 20.3% (98/483) in a maximum care clinic or university hospital, 20.1% (97/483) in a clinic with focussed care, 17.8% (86/483) in a basic and standard care clinic, 7.2% (35/483) in a rehabilitation clinic and 5.2% (25/483) in another type of facility. In total 77.8% (368/473) had the clinical focus on orthopedic surgery, 56.7% (268/473) on trauma surgery, 13.7% (65/473) on sports medicine and 7.2% (34/473) on rehabilitative medicine.

Among the residents, 25.5% (24/55) were female and 72.7% (40/55) were male (1 abstention) and the average age was 35.0 years (SD: ±5.7 years). Of the residents 40.0% (22/55) worked in a maximum care clinic or university hospital, 25.5% (14/55) worked in a clinic with focussed care or a basic and standard care clinic. On average, the residents were in their 5th year of residency (mean: 4.9 years; SD: ±1.7 years).

### Self-assessed competences in the manual evaluation of different body regions

Most participants (83.1%, 369/444) stated that they agree or rather agree to have a good general understanding of non-operative treatment options, while only 0.68% (3/444) rather disagreed or disagreed and 4.95% (22/444) were neutral (mean score 0.81; SD: ±0.22). Among residents 35.4% (17/48) agreed or rather agreed with this statement and the mean score was 0.56 (SD: ±0.25).

Similarly, 81.0% (359/443) of all participants stated that they feel confident to apply non-operative treatment options in their daily clinical routine and only a minority disagreed or rather disagreed (mean score 0.81; SD: ±0.25). Among residents 41.7% (20/48) agreed or rather agreed to this statement with a mean score of 0.53 (SD: ±0.29).

**Fig. 1:**
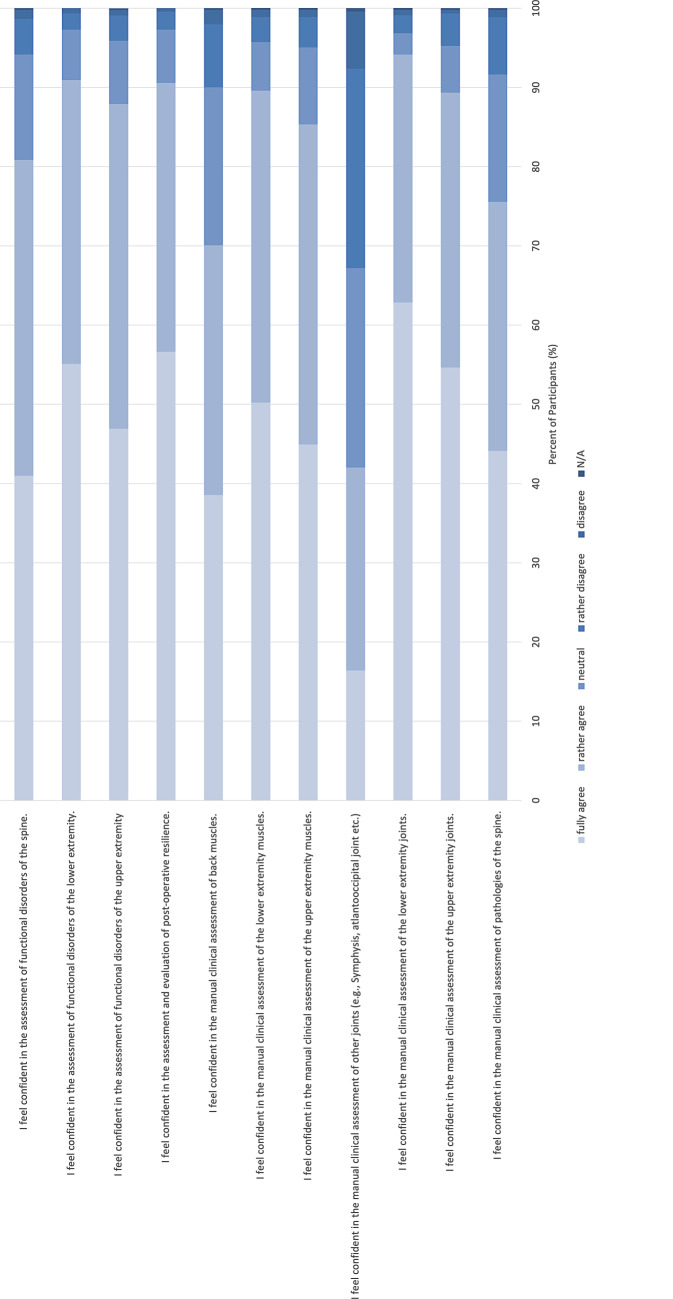
Self-assessed competences in the manual evaluation of different body regions

**Fig. 2:**
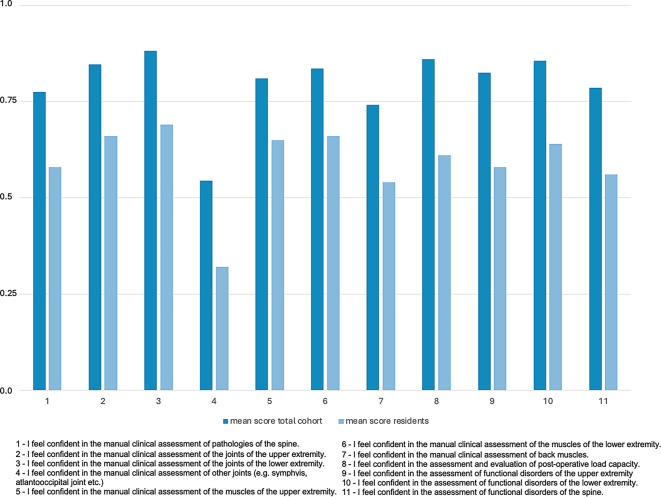
Mean Likert scale scores (where 1.0 = “I totally agree”, 0.75 = “I rather agree” (0.75), 0.5 = “neutral”, 0.25 = “I rather disagree” and 0.0 = “I totally disagree”) of the self-assessed competences in the manual evaluation of different body regions for the total cohort and residents

An overall full analysis of the self-assessed competences in the manual evaluation of different body regions is presented in Fig. 1. The comparison of the mean scores between the overall and the resident cohort is presented in Fig. 2.

### Self-assessed non-operative diagnostic and treatment competences

The highest self-assessed non-operative diagnostic and treatment competences were knowledge on the application of splints and casts (mean: 0.83; SD: ±0.21), postoperative physiotherapy, ergotherapy and sports therapy (mean: 0.83; SD: ±0.22), physiotherapy, ergotherapy and sports therapy after acute trauma (mean: 0.82; SD: ±0.22) and medication-based pain therapy (mean: 0.82; SD: ±0.20). Similarly, the highest self-assessed non-operative diagnostic and treatment competences among residents were knowledge on medication-based pain therapy (mean: 0.72; SD: ±0.26), the application of splints and casts (mean: 0.66; SD: ±0.24) and postoperative physiotherapy, ergotherapy and sports therapy (mean: 0.61; SD: ±0.26).

The lowest self-assessed non-operative diagnostic and treatment competences were knowledge on acupuncture (mean: 0.37; SD: ±0.38), magnetic field therapy (mean: 0.32; SD: ±0.34) and nutritional aspects and supplements after trauma (mean: 0.28; SD: ±0.31). Among residents the lowest self-assessed competences were magnetic field therapy (mean: 0.15; SD: ±0.20), acupuncture (mean: 0.21; SD: ±0.32) and nutritional aspects and supplements after trauma (mean: 0.29; SD: ±0.28).

**Fig. 3:**
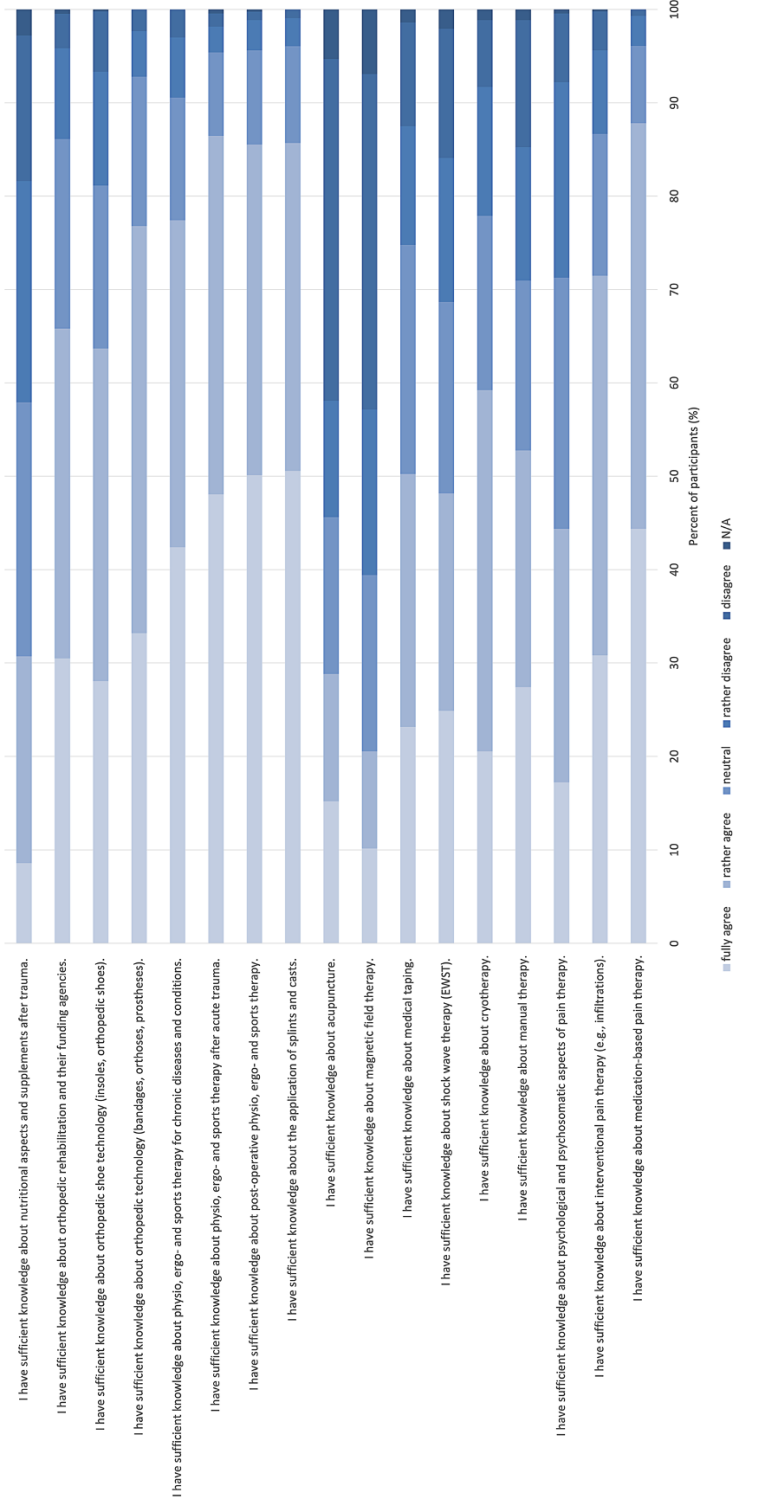
Self-assessed competences in different non-operative diagnostic and treatment possibilities

**Fig. 4:**
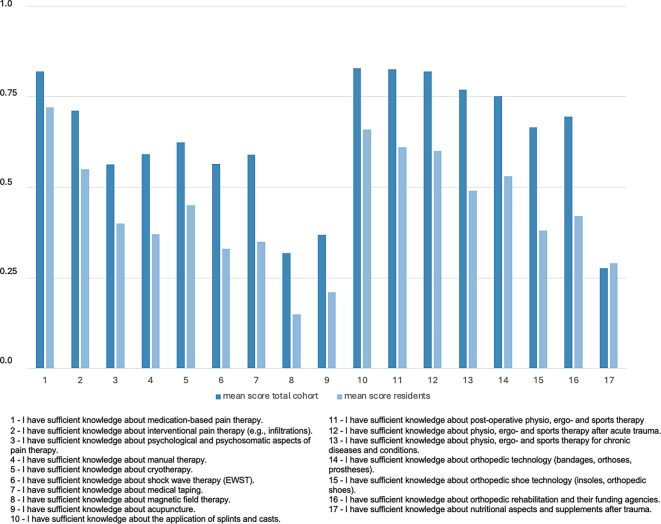
Mean Likert scale scores (where 1.0 = “I totally agree”, 0.75 = “I rather agree” (0.75), 0.5 = “neutral”, 0.25 = “I rather disagree” and 0.0 = “I totally disagree”) of the self-assessed competences in different non-operative diagnostic and treatment possibilities for the total cohort and residents

A full analysis of the self-assessed competences in different non-operative diagnostic and treatment possibilities is presented in Fig. 3. The comparison of the mean scores between the total sample and residents are presented in Fig. 4.

### Scope of non-operative education experienced

Most of the respondents (78.4%, 330/421) stated that their clinic actively educates non-operative treatment methods and only 18.3% (77/421) stated that the clinic does not educate non-operative treatment options and 3.3% (14/421) were unsure.

In total 83.2% (351/422) stated that they have already taken part in further training that focusses on non-operative treatment options. Only 15.6% (66/422) have never taken part in such training. Overall, 49.86% (179/359) have taken part in further training for manual therapy, 22.56% (81/359) for sports medicine and 9.47% (34/359) for kinesiological taping. Among residents 63.0% (17/27) have taken part in further training for manual therapy, 18.5% (5/27) for sports medicine and 18.5% (5/27) for kinesiological taping.

In contrast 77.7% (328/422) of the respondents stated that their clinic does not promote research projects in the field of non-operative treatment options.

Furthermore, only a minority of the respondents (27.1%, 114/420) spent part of the residency training in the outpatient sector and only 18.0% of the respondents stated that the clinic offers rotation into non-operative sections. Moreover, 61.8% (230/372) stated the clinic does not have fixed rotation into non-operative sections and 20.2% (75/375) were not sure.

### Requests concerning non-surgical education

Overall, 84.2% (320/380) of the respondents stated that they wish that there is an increased focus on non-operative treatment options in the field of orthopedic and trauma surgery, while 8.2% (31/380) were unsure.

Among residents, 82.5% (33/40) would favor a rotation in the outpatient sector, while only 7.5% (3/40) did not want such a rotation and 10.0% (4/40) were not sure. The mean duration preferred for such a rotation among residents was 7.7 months (*n* = 40, SD: ±4.2 months, min.: 1 month, max.: 24 months). Similar results can be displayed for specialized physicians who are not in leading positions. Here, 72.2% wished for a rotation in the outpatient sector, 23.5% did not wish for such a rotation and 11.3% were not sure.

Additionally, among residents 57.5% (23/40) would favor a rotation to a rehabilitation facility, while 30.0% (12/40) did not want such a rotation and 12.5% (5/40) were not sure. The mean duration wished for such a rotation among residents was 5.6 months (*n* = 40, SD: ±3.0 months, min.: 0 months, max.: 12 months). Similar results can be found for specialized physicianswho are not in leading positions, 58.6% wished for such a rotation, 30.2% did not and 11.2% were unsure.

## Discussion

This study aimed to present the current state of the conservative, non-surgical knowledge among all levels of expertise of orthopedic and trauma surgeons in Germany. To the authors’ knowledge this is the first study of this scope on this topic.

First, the study in general indicates that the respondents subjectively have a good self-assessed general knowledge and understanding about non-operative treatment options and feel confident in the manual clinical assessment of the musculoskeletal system. Interestingly the self-assessed knowledge on the manual clinical assessment of the joints, muscles and functional disorders was highest for the lower extremities, followed by the upper extremities and the spine. More precisely, for the lower extremities the clinical assessment of the joints showed a mean score of 0.88, the assessment of the muscles displayed a mean score of 0.84 and the assessment of functional disorders showed a mean score of 0.86. For the upper extremities the clinical assessment of the joints showed a mean score of 0.85, the assessment of the muscles displayed a mean score of 0.81 and the assessment of functional disorders showed a mean score of 0.82; however, in contrast to this the manual clinical assessment of the spine displayed lower mean scores than for the upper and lower extremities. Here, the manual clinical assessment of pathologies of the spine had a mean score of 0.78, the assessment of back muscles of 0.74 and the assessment of functional disorders of the spine 0.79. This is particularly interesting when considering that like in other western countries, back and neck pain is a common condition, which can have a considerable influence on a patient’s mobility and quality of life. In a German telephone survey performed by the Robert Koch Institute 61.3% of the respondents reported back pain in the last 12 months [7] and 15.5% stated that they have chronic back pain [7]. In Germany back pain is one of the most common reasons for using the healthcare system, taking sick leave and for early retirement [7]. The appropriate diagnostics and treatment are essential for the management of back pain. A very important pillar, especially for unspecific acute back pain is conservative, non-operative treatment [8]. Knowing that, a more focused education of non-surgical diagnostic and treatment options regarding the spine should be focused on. At this point it must be noted that the German Spine Society (Deutsche Wirbelsäulengesellschaft, DWG) has launched a course series with basic and advanced courses on the conservative, non-operative treatment of conditions of the spine. These courses contain topics like manual therapy, pain management and psychosomatic aspects of conditions of the spine.

Secondly, the self-assessed competences related to different non-operative diagnostic and treatment methods varied widely among the respondents. The highest self-assessed competences were knowledge on the application of splints and casts, postoperative physiotherapy, ergotherapy and sports therapy, physiotherapy, ergotherapy and sports therapy after acute trauma and medication-based pain therapy. These also represent the most common and most widely used competences for a wide range of orthopedic and traumatological diseases. On the other hand, the respondents only indicated neutral understanding in competences like manual therapy, psychological and psychosomatic aspects of pain therapy and interventional pain therapy. Here, improvement should be achieved. The lowest competences were knowledge on acupuncture, magnetic field therapy and nutritional aspects after trauma. This is not surprising as these methods are not reflected in the current German medical training regulations. The great range of competences might, however, be an indication for a missing education of non-operative knowledge and skills on curriculum, didactic and organizational levels. Summing up, for the most common competences there is a subjectively well-established knowledge which can be improved for manual therapy as well as psychological and psychosomatic aspects of pain therapy and interventional pain therapy to further improve the conversative treatment options for patients.

Third, the results suggest that a considerable percentage of the respondents are interested in non-operative content within the field of orthopedic and trauma surgery and desire an increased focus on it in further education. The results show that there is a considerable interest by resident physicians and specialized physicians who are not in leading positions in organized rotation into the outpatient sector and into rehabilitation facilities to acquire more knowledge and skills in non-operative treatment options. More precisely, 82.5% of resident physicians and 72.2% specialized physicians who are not in leading positions would like to have a rotation to the outpatient sector. Moreover, 57.5% of resident physicians and 58.6% of specialized physicians who are not in leading positions would like to have a rotation into a rehabilitation facility.

It is noteworthy that within this cohort more than 60.0% of the respondents stated that their institution offers such rotations and represents a positive result of our study. These abovementioned findings could underline a significant interest in pursuing additional training in non-operative treatment methods through specialized rotation. These rotations could be of particular interest especially in view of the currently planned shift tp outpatient treatment of the German healthcare system. Ambulatory settings provide exposure to a wide range of orthopedic conditions that may not be as prevalent in the clinical setting. Residents can therefore gain experience in diagnosing and managing chronic conditions and overuse injuries which are mainly treated through conservative approaches. Furthermore, ambulatory rotation could emphasize the importance of preventive measures and rehabilitation strategies, including physical therapy, exercise prescription and patient education. Lastly working in ambulatory care settings could enable residents to have a better understanding of the broader ambulatory healthcare system, including insurance processes, referrals, and collaborative care with primary care physicians and other specialists. This knowledge is crucial for providing comprehensive patient care.

Similarly, according to the respondents educational institutions do not generally promote research projects that investigate non-operative treatment options. More than 75.0% of the respondents stated that their clinic does not promote research projects in the field. It must however be critically noted that it was not inquired whether the institution promotes research in general. Research provides the evidence base for medical practice. By conducting studies on conservative treatment methods, clinicians can determine the effectiveness of various interventions. This evidence helps guide orthopedic and trauma surgeons in making informed decisions about the most appropriate and successful treatment for specific conditions, which could be non-surgical or surgical. Research in this area helps identify situations where surgery might be avoided or delayed, minimizing the potential risks and complications associated with surgical procedures.

The presented study has certain limitations. First, surveys have minor levels of evidence in general and their outcome can be affected by the participants’ understanding of the questions. In addition, due to the voluntary participation, orthopedic and trauma surgeons with a more critical attitude towards or less interest in the topic might be underrepresented in the study, posing a potential bias. Moreover, the study should be interpreted with caution because it focusses on the self-assessed and therefore the subjective assessment of the respondents. While filling out the survey the theoretical knowledge might be overestimated in comparison to the actual practical ability. In the future, objective assessments of non-surgical methods might be of interest. Furthermore, the number of participants cannot be taken as representative for all orthopedic and trauma surgeons in Germany and it must be noted that there were imbalances in the gender, age, and educational levels of the respondents. Particularly, residents were underrepresented in the study with only 11.5%. Therefore, residents were analyzed separately in this study because it provides targeted insights into the unique needs and challenges, enabling more effective and tailored educational programs and policies. Nonetheless, the study provides a good first insight into the current state of conservative, non-surgical knowledge among orthopedic and trauma surgeons in Germany and the presented results offer a foundation for further research discussions and potential actions to address the identified needs and gaps in education and training in the field.

## Conclusion

Orthopedic and trauma surgeons in Germany subjectively have solid self-assessed knowledge on conservative, non-surgical diagnostic and treatment options; however, improvement can be achieved for the upper extremities as well as the spine, especially because the latter has a high impact on the German healthcare system. Moreover, rotation into the ambulatory sector and rehabilitation facilities are desired and could further improve the non-surgical skills.

## Data Availability

The datasets used and/or analyzed during the current study are available from the corresponding author on reasonable request.
